# Surgery

**Published:** 1897-04

**Authors:** John Parmenter

**Affiliations:** Professor of anatomy and adjunct professor of clinical surgery, Buffalo University Medical College


### Progress in Medical Science.

### SURGERY.

CONDUCTED BY JOHN PARMENTER, M. D.,

Professor of anatomy and adjunct professor of clinical surgery, Buffalo University Medical College.

### NONOPERATIVE TREATMENT OF APPENDICITIS.

FOWLER (*New York Polyclinic*, December 15, 1896,) discusses the question of the nonoperative treatment of appendicitis. Various causes may lead to the substitution of medical for surgical treatment in this disease. These include unsuitable environment for operation, absence of skilled surgical aid, refusal to permit operation, bad general condition of the patient due to other complications, or to a too advanced state of the disease. Speaking of simple uncomplicated appendicitis, the author believes that "with the observance of the principles of aseptic surgery many practitioners possess sufficient anatomical knowledge and surgical skill to carry out an operation for excision of the appendix early in the affection," but many cases die yearly from lack of courage and confidence in his ability on the part of the physician ; on the other hand, many cases require the highest skill on the part of the surgeon and of his assistant. Hence, for one reason or another, operative procedures are not adopted. The author then recommends that the following line of treatment be adopted : The patient must be kept absolutely quiet in the recumbent posture, not being allowed to leave the bed for any purpose whatever ; all movements should be passive so far as possible, such motions as even turning in bed or sitting up should be done quietly and slowly. The bowels should be regulated by first giving an enema, and if the disease is seen early an adequate dose of Epsom salts should be administered. When cases are seen for the first time later in the disease, it is better to wait three or four days for adhesions to occur and then secure a movement of the bowels with an enema. No cathartics should be employed at this time ; no straining on the part of the patient should be permitted. All these precautions tend to avoid rupture of adhesions and escape of infectious material.

Regarding the administration of opium, the author declares it to be "a two-edged sword that cuts both ways, and unfortunately its keenest edge is turned in the direction of the patient's comfort." He further says : "When the diagnosis has been made, and if the attendant is fully cognisant of the fallacies in the prognosis which this drug gives rise to, and is on his guard against being misled by the apparent improvement in the symptoms which follow its administration, the employment of the drug is justifiable, in the event of a failure of other measures to relieve pain and restlessness. If the question of operating or not operating depends upon the improvement or otherwise, since the drug cannot save the patient, but only ameliorate his symptoms, it should be withheld. If improvement is noted and no opium has been given, there can be but slight opportunity for error, particularly if the symptom of localised tenderness be carefully watched and noted at its full value. With the patient under the influence of opium, it is not possible to intelligently discuss the question of operation, if this is to turn upon an accurate estimate of the patient's condition, for the reason that it is not possible to form such estimate ; neither can it be known whether convalescence is assured or a catastrophe impending."

The writer believes that treatment by drugs alone is equivalent to relying upon nature, and that when used the main indications are to relieve suffering and to protect, as far as possible, against perforative and infectious peritonitis and general septic infection. Therefore, opium is to be used in the smallest dose necessary to keep the patient approximately comfortable. He says of it : "If carefully watched it may be employed. It must be confessed, however, that in spite of the general prevailing opinion upon this point it is more difficult to state the indications than the contra-indications for its use."

Local applications are of doubtful value. They may be used either hot or cold, according to the comfort of the patient and the effect produced. Nutrition must be maintained by appropriate food. The skin and kidneys are to be kept functionally active. Antipyretics are usually contra-indicated, for the reason that they mask the disease and cannot benefit a fever which is septic in origin. Tonic doses of quinine may be given. Strychnia in hypodermatic doses of grain 1-30 is useful. Spartein (grain $\frac{1}{4}$), combined with nitroglycerine and caffeine, assists renal elimination by increasing the blood pressure.

When an appendical abscess or an intraperitoneal encapsulated suppurative collection ruptures, the treatment varies with the route taken by the escaped material.

Perforative peritonitis being usually fatal, the treatment resolves itself into producing euthanasia, with anodynes used hypodermatically on account of the early and persistent vomiting which characterises this condition. When the discharge is into the intestinal canal, the diarrhea is not to be checked ; when into the bladder, treat the resulting cystitis ; when into the vagina, use vaginal douches and apply an antiseptic pad ; when into the pleural cavity, the pus must be evacuated or the patient dies miserably. This may also be the case where the pus escapes into the posterior peritoneal connective tissues of the back or through the pelvis down into the thigh or into the layers of the abdominal wall.

Septic hepatitis and pyemia may occur, and while always grave, recovery may take place.

The author concludes his article with the expressed belief that, with the exception of the cases that are nonprogressive in character for the first twenty-four hours, and which recover after simple rest in bed and the exhibition of a saline, cases of appendicitis that recover under so-called medical treatment do so from the vis medicatrix naturæ, to a large extent, and that the efforts of the physician will be directed toward assisting nature in walling off the septic material.

---

### FULMINATING APPENDICITIS.

CRUTCHER (*International Journal of Surgery*, January, 1897,) contributes an interesting article upon fulminating appendicitis, which begins with the expressed opinion of several of the leading surgeons of America upon two practical points put in the form of interrogatories—namely :

1. Do you know of any ordinary means by which perforation can be foretold ?

2. What have been your results in fulminating appendicitis ?

The answers are unanimous to the effect that perforation cannot be foretold with any certainty and that operation is practically useless. After relating cases of his own in which the typical symptoms of general peritonitis have been present, the author goes on to state that shock is the most reliable guide in appendicitis and that the pain, the pulse, the temperature and other symptoms are not accurate enough to be scientifically classified. He says of shock that it tells "a pretty accurate story and its meaning in appendicitis cannot be mistaken. Unfortunately, in many cases the landmarks of shocks are too obscure to afford much help in reaching a conclusion." He sums up his article with the following statements:

*Pain.*—If violent, sudden and persistent, it indicates probable seriousness of the attack, but indicates nothing as to the natural defenses for limiting infection. On the contrary, absence of pain is undecisive between gangrene and resolution.

*Pulse and Temperature.*—While a rapid pulse and high temperature favor the destructive process, their absence is no assurance of recovery. Referring to this, Tyson remarks that "too much stress cannot be laid upon the fact that there may be gangrenous appendicitis in the presence of normal temperature."

*Shock.*—The presence of shock, if undoubted, is a very grave symptom, generally indicating perforation. On the contrary, the most deadly attacks often occur without it.

*Sensitiveness.*—If persistent and highly developed, generally indicates destructive inflammation, but gives no clue concerning the limitations of infection.

*The Expression.*—Of no material value before the development of grave conditions.

*Perforation.*—This can no more be foretold than the perforation of typhoid or the rupture of an aneurism.

My own practice is to save the patient first by operating upon nonseptic tissues, if possible, and to argue the case afterward.

---

### SURGICAL TREATMENT OF PERFORATING TYPHOID ULCER.

FINNEY (*Annals of Surgery* for March, 1897,) contributes an article on The surgical treatment of perforating typhoid ulcer, which contains an excellent résumé of this now important subject. He devotes some space to the early history of the condition and collects from the literature on the subject some forty-six cases, adding to this number six hitherto unpublished cases, three of which were his own. Our present knowledge relating to the conditions under which perforation of the ulcerated Peyer's patches occurs is briefly reviewed and the following conclusions deduced: Perforation of the intestine occurs in from 1 to 2 per cent. of all typhoid cases. As a cause of death in fatal cases it is found in between 6 and 7 per cent. It occurs in the ileum, usually within the last two feet, in over 80 per cent. of all cases; in the large intestine, in more than 12 per cent.; in the appendix vermiform, in about 5 per cent. of all cases. It is usually single, but may be multiple. No definite relation between perforation and severity of the attack has been noted. It occurs in mild as well as severe cases. The symptoms marking the onset may be sudden and severe, very mild or entirely wanting. It occurs oftener in men than women, the ratio being three to one. It is rarely met with in children. It occurs most frequently between 20 and 30 years of age and in the third week of the disease. The duration of life following perforation is, as a rule, short. The statistics of Fitz are quoted, which give over 37 per cent. dying on the first day, over 29 per cent. on the second day, and over 83 per cent. of all cases during the first week. The perforation is always followed by a suppurative peritonitis, general or localised. When localised, the termination is much more favorable. Recovery after intestinal perforation has occurred and, therefore, the accident, while always severe, need not prove fatal in every case. The fact is noted in this connection that peritonitis, which often proves fatal, may develop in the course of typhoid fever without any discoverable perforation of the intestine, which means that peritonitis may occur from other causes than perforation, such as rupture of softened mesenteric glands, abscesses of the liver, perforating ulcers of the gall bladder, softened infarcts of the spleen, and the like. In this connection the author quotes Fitz, for he has very aptly remarked: "Since perforation of the intestine in typhoid fever may take place without any suggestive symptoms, and since suggestive, even so-called characteristic, symptoms may occur without any perforation having taken place, it must be admitted that recovery from such symptoms is no satisfactory evidence of recovery from perforation."

It is, therefore, impossible to estimate the true percentage of recoveries from perforated typhoid ulcers without operation, and of deaths where no autopsy has been made.

The author then gives some statistical data regarding the cases collected which need not be reproduced here, but which deserve the attention of all persons interested in the subject. The author's conclusions may, however, be summarised:

The age and sex bear out pretty closely their respective percentages as quoted in the beginning of the paper.

The third week is by far the most frequent time for the occurrence of perforation. It occurred more frequently in mild than in severe cases. The most constant and characteristic sign of perforation recorded was sudden, severe abdominal pain, persisting with increasing intensity for some time. Nausea and vomiting were present in about 50 per cent. of the cases.

How long the time between perforation and the operation affected the result does not appear. Some cases operated upon early died the soonest, and vice versa. However, both reason and the little evidence deduced from these cases bear out the presumption that the best time for operation is as early as possible following perforation, and after the patient has recovered somewhat from the primary shock and collapse. The operation should be thorough and all perforations noted. Time will be saved by examining the last two feet of the ileum and noting the condition of the cecum and appendix.

Irrigation is not considered as safe as wiping the peritoneal cavity. The perforations are dealt with by bringing the perforated part out of the abdomen and leaving it there for subsequent resection and anastomosis after the patient has recovered from the fever. The edges of the ulcers where small and where the surrounding tissues are healthy are sutured without previous excision of the edges, the evidence being that union of the opposed peritoneal surfaces takes place as satisfactorily in cases where the edges of the ulcers are not excised as in those which have been so treated.

Drainage should always be employed. In general septic peritonitis the contents of the peritoneal cavity should be treated by evacuation of its septic contents and then by the establishment of effective drainage, which is best accomplished by means of wicks of bismuth or iodoform gauze carried into the different parts of the cavity and their ends brought out through the abdominal wound, which can be tightly sutured throughout the rest of its extent. Drainage in the most dependent portion should be practised more often than it is.

The question of differential diagnosis is then taken up and particular attention given to the marked leucocytosis occurring at the time of perforation.

Medicinally, if stimulation is necessary, reliance is to be placed on hypodermics of strychnia, enemata of several ounces of hot black coffee and the transfusion into the cellular tissues under the breast of a quart or more of the normal salt solution. When there is such distension the bowels should be moved early and thoroughly by calomel in broken doses, followed by salts, and, if necessary, a high turpentine and soap suds enema.

The writer sums up his impressions as follows :

"(1) Of all the so-called diagnostic signs of perforating typhoid ulcer most reliance is to be placed upon the development of an attack of severe, continued abdominal pain, coupled with nausea and vomiting, and at the same time a marked increase in the number of white blood corpuscles.

"(2) The surgical is the only rational treatment of perforating typhoid ulcer.

"(3) There is no contra-indication to the operation, surgically speaking, save a moribund condition of the patient."

